# Registered therapeutic trials in adults with functional dyspepsia: a scoping review of design and registration characteristics

**DOI:** 10.3389/fmed.2026.1956161

**Published:** 2026-10-14

**Authors:** Kangmeng Yao, Yixuan Lang, Wei Deng, Shuo Zhang, Sheng Liu, Yan Leng

**Affiliations:** 1 College of Traditional Chinese Medicine, Changchun University of Chinese Medicine, Changchun, China; 2 Department of Liver, Spleen and Stomach Diseases, The Affiliated Hospital to Changchun University of Chinese Medicine, Changchun University of Chinese Medicine, Changchun, China

**Keywords:** clinical trials, functional dyspepsia, scoping review, trial design, trial registration, WHO ICTRP

## Abstract

**Background:**

Functional dyspepsia (FD) affects quality of life and healthcare use. Previous studies have examined individual aspects of FD trial methodology, but a broad account of registered therapeutic trials is still needed. This scoping review mapped their design and registration characteristics to inform future trial design and reporting.

**Methods:**

WHO International Clinical Trials Registry Platform (ICTRP) was searched on 4 September 2026 for registered therapeutic interventional trials in adults with FD. Records referring to the same trial were linked into one trial unit. Two reviewers independently screened trial groups and assessed trial-level eligibility. Corresponding source registries were checked when ICTRP information was missing, incomplete, or inconsistent. Analyses were descriptive.

**Results:**

The review included 421 underlying registered trials, of which 191 (45.4%) were first registered during 2021–2026. Randomized allocation was recorded in 315 trials (74.8%), and 324 (77.0%) used parallel-group designs. Registration preceded the recorded study start date in 252 trials (59.9%). An explicit Rome diagnostic version was identified in 278 trials (66.0%). Primary outcomes were specified in 417 trials (99.0%), with FD symptoms represented in 374 (88.8%). A named primary FD symptom instrument was identified in 166 trials (39.4%). A safety or adverse-event outcome was identified in 103 trials (24.5%), intervention duration in 149 (35.4%), and post-intervention follow-up duration in 15 (3.6%).

**Conclusion:**

Registered FD trials cover a range of pharmacological and non-pharmacological interventions, but the documentation of eligibility criteria, masking, symptom measures, safety-related outcomes, intervention duration, and post-intervention follow-up varies. Earlier registration and clearer reporting of these elements would help readers interpret trial designs and compare studies.

## 1 Introduction

Functional dyspepsia (FD) is a disorder of gut-brain interaction characterized by postprandial fullness, early satiation, epigastric pain, and epigastric burning, without structural disease sufficient to explain the symptoms. Under the Rome IV framework, FD is classified as postprandial distress syndrome (PDS), epigastric pain syndrome (EPS), or an overlap of both. Population-based studies show that FD is common worldwide and affects quality of life and healthcare use (1–3).

Treatment options include acid suppression, prokinetics, central neuromodulators, and non-pharmacological approaches (2, 3). This range reflects the complex pathophysiology of FD and the absence of a universally effective treatment (2, 3). The interpretation of treatment effects depends in part on how trials define their populations, handle overlapping conditions, select outcomes, apply masking, and specify treatment and follow-up periods (4, 5).

FD trial methodology has been examined for several decades. A systematic analysis of drug-trial methods was published in 1996, followed by Rome-associated guidance on treatment trials in functional gastrointestinal disorders (4–6). Later studies addressed endpoint selection and patient-reported outcome measures (7–9). These contributions provide guidance on particular aspects of trial design, but do not offer a contemporary overview of design and registration characteristics across registered FD therapeutic trials.

Trial registries provide a structured source for examining these characteristics. We therefore conducted a scoping review of registered therapeutic interventional trials in adults with FD worldwide. We examined registration patterns, trial design, intervention categories, diagnostic and eligibility criteria, outcome measures, and the availability of key registry information.

## 2 Methods

### 2.1 Review design, scope and protocol

This scoping review examined registered therapeutic interventional trials in adults with functional dyspepsia (FD). The underlying registered trial was the unit of analysis. A scoping-review approach was chosen to describe the range of interventions and map design and registration characteristics across several methodological domains.

The review question and eligibility criteria followed the Population-Concept-Context (PCC) framework (10, 11). The Population was adults with explicitly defined FD, including the historical term non-ulcer dyspepsia (NUD) and the subtypes postprandial distress syndrome (PDS) and epigastric pain syndrome (EPS). The Concept was the design and registration characteristics of therapeutic interventional trials intended to improve FD-related clinical outcomes. The Context was unrestricted by country, care setting, intervention type, sponsor, or recruitment status. We asked: What design and registration characteristics are observed among registered therapeutic interventional trials in adults with functional dyspepsia worldwide?

No review protocol was prospectively registered or published. The operational protocol and standardized decision rules were finalized before the WHO ICTRP search, screening, trial linkage, and data extraction; the full protocol is provided in Supplementary Protocol S1. The review was guided by JBI scoping-review methodology (10, 11). Reporting followed PRISMA-ScR (12), and the search methods were documented using PRISMA-S (13).

### 2.2 Information sources and search strategy

Trials were identified through trial registries. The WHO International Clinical Trials Registry Platform (ICTRP) was the sole discovery source because it provides a single portal for records from multiple international registries (14).

Before the search was run, the strategy was independently reviewed by a second author who had not helped develop it. A Standard Search was conducted on 4 September 2026 using the following query:

dyspepsia OR “postprandial distress syndrome” OR “post prandial distress syndrome” OR “epigastric pain syndrome”

No filters or limits were applied. The search retrieved 1,027 registry records, which were grouped by ICTRP into 993 trial groups.

When ICTRP information was missing, incomplete, or inconsistent, the corresponding source registry was checked. Verifiable source-registry information was used to supplement missing fields or resolve discrepancies. When ICTRP and source-registry information differed or records had been updated, we prioritized the most recent verifiable source-registry record available at the time of data verification, except for target sample size (Section 2.4). For fields resolved under this rule, the extracted values describe the latest verifiable registry information available at data verification, which does not necessarily represent the version in place before participant enrollment.

### 2.3 Eligibility criteria and study selection

Eligible studies were registered therapeutic interventional trials in adults aged 18 years or older with explicitly defined FD, NUD, PDS, or EPS. The intervention had to be intended to treat or improve FD, and the adult FD treatment population had to be independently identifiable. This requirement also applied to mixed-age and mixed-diagnosis studies. Trials involving FD with overlapping gastroesophageal reflux disease (GERD) or irritable bowel syndrome (IBS) were eligible when FD was explicitly defined and the FD treatment population could be independently identified.

Trials had to prespecify at least one FD-related clinical treatment outcome, whether primary or secondary. Eligible outcomes included FD symptom severity or response, global symptom improvement, FD-related quality of life, or another explicitly defined clinical treatment outcome.

Trials were excluded if they were observational or non-interventional; involved only children or healthy volunteers; evaluated diagnostic, screening, validation, provocation, or purely mechanistic procedures without therapeutic clinical intent; involved organic or secondary dyspepsia without a separately identifiable FD population; or referred only to nonspecific dyspepsia or related upper gastrointestinal symptoms without sufficient registry evidence to establish FD. We also excluded trials that primarily treated another disorder with FD considered only as a secondary outcome or ancillary analysis, mixed-population trials in which the FD treatment population could not be identified separately, and records with insufficient information to confirm eligibility. Trials with only physiological, imaging, biomarker, microbiome, pharmacokinetic, pharmacodynamic, or other mechanistic outcomes, and no prespecified clinical FD treatment outcome, were excluded.

Helicobacter pylori eradication trials were eligible when the population was explicitly defined as FD or NUD and at least one FD-related clinical treatment outcome was prespecified. Trials limited to eradication, antimicrobial resistance, or other non-FD outcomes were excluded.

Two reviewers (KMY and YXL) independently screened the ICTRP trial groups using the same eligibility criteria. Potentially eligible or uncertain records were retained for trial linkage and further review. After trial linkage and de-duplication, the remaining underlying trial units underwent independent eligibility assessment by the same two reviewers. Disagreements were resolved through discussion and, when necessary, adjudication by a third reviewer (SL). Exclusion reasons were recorded using standardized categories. The selection process was summarized in a PRISMA-ScR flow diagram that distinguished retrieved registry records, ICTRP trial groups, linked trial units, exclusions, and included trials.

### 2.4 Trial linkage, data charting and variable harmonization

Records referring to the same underlying trial were linked and collapsed into one trial unit before analysis. Linkage relied primarily on explicit primary or secondary registration identifiers and direct cross-registry references. When these were unavailable, records were compared using the title, sponsor, intervention and comparator, target sample size, country or study sites, recruitment or start dates, and study design. Title similarity alone was insufficient for merging. Records were kept separate when the available evidence did not show that they represented the same trial. KMY performed the linkage, YXL checked the decisions, and SL was available to adjudicate unresolved disagreements. Supplementary Protocol S1 provides the detailed linkage rules.

Data were charted independently at the trial level by two reviewers (WD and SZ) using a standardized extraction form. Disagreements were resolved by discussion or, when necessary, adjudication by a third reviewer (SL).

Extracted variables included source registry; registration date and recorded study start date; recruitment status; country; sponsor; study design; allocation; intervention model; control type; number of arms; masking roles; target sample size; registered centers or study locations; intervention category; FD diagnostic criteria and subtype; upper gastrointestinal endoscopy requirements; Helicobacter pylori criteria; GERD and IBS overlap rules; baseline symptom thresholds; intervention duration; post-intervention follow-up; registered primary and secondary outcome domains; named symptom instruments; and explicit safety or adverse-event outcomes. Equivalent country or region names were harmonized before geographic summaries were generated. The variable definitions and classification rules are outlined in Supplementary Protocol S1.

For registry-source summaries, each trial was classified by the source registry of its earliest linked registration. All linked records remained available for verification and supplementation of trial information. For target sample size, we classified a positive planned enrollment target as confirmed only when it could be verified in ICTRP or a linked source registry. When linked records reported different positive targets, we retained the value from the record with the earliest registration date. Conflicts on that earliest date and current enrollment figures that could not be identified as planned were left unconfirmed (Supplementary Protocol S1, Section 9.8).

Masking was harmonized by explicitly reported roles because terminology differed across registries. Masking status was recorded separately for participants, care providers or interventionists, investigators, and outcome assessors. Generic single-, double-, triple-, or quadruple-blind labels were not used to infer unreported roles. Open-label status was recorded only when the registry explicitly stated that the trial was open-label or used no masking. For each role, “Yes” and “No” were used when masking status could be determined from the record; “Unclear” when masking was reported but the role assignment could not be resolved; and “Not specified” when no usable masking information was available. Masking was summarized descriptively, with its feasibility considered in relation to intervention type, and was not used as a stand-alone measure of methodological quality.

Missing or unresolved registry information was retained as such. It was not taken as evidence that a procedure, assessment, or follow-up had not been performed. For endoscopy, “not mentioned” was used when relevant eligibility information was available but no endoscopy requirement was stated; “insufficient information” was used when the record could not be classified.

### 2.5 Statistical analysis

Analyses were descriptive and used the underlying registered trial as the unit of analysis. Categorical variables were summarized as frequencies and percentages. Continuous or ordered variables were grouped where appropriate; confirmed target sample sizes were also summarized using the median, interquartile range, and range. Unless otherwise stated, percentages used all included trials as the denominator, with not-specified categories retained.

Outcome domains were not mutually exclusive, so their percentages could sum to more than 100%. Temporal patterns were summarized by the earliest linked registration year. Trials involving more than one country or region contributed to each applicable location; country-specific percentages were therefore also non-mutually exclusive. Analyses were performed in Microsoft Excel using the final trial-level dataset. No formal risk-of-bias or methodological-quality appraisal, meta-analysis, or inferential comparison of treatment effects was performed.

## 3 Results

### 3.1 Study selection and formation of the trial-level dataset

The WHO ICTRP search retrieved 1,027 registry records, organized by ICTRP into 993 trial groups. Initial screening retained 549 groups for further review and de-duplication. Linking records that referred to the same trial left 543 underlying registered trials for final eligibility assessment. Six underlying trial units were formed by linking ICTRP trial groups without explicit shared registration identifiers on the basis of concordant trial characteristics. No trial-linkage disagreement required adjudication by the third reviewer (SL). Following independent assessment and adjudication, 421 trials were included and 122 were excluded. Figure 1 shows the selection process.

**Figure 1:**
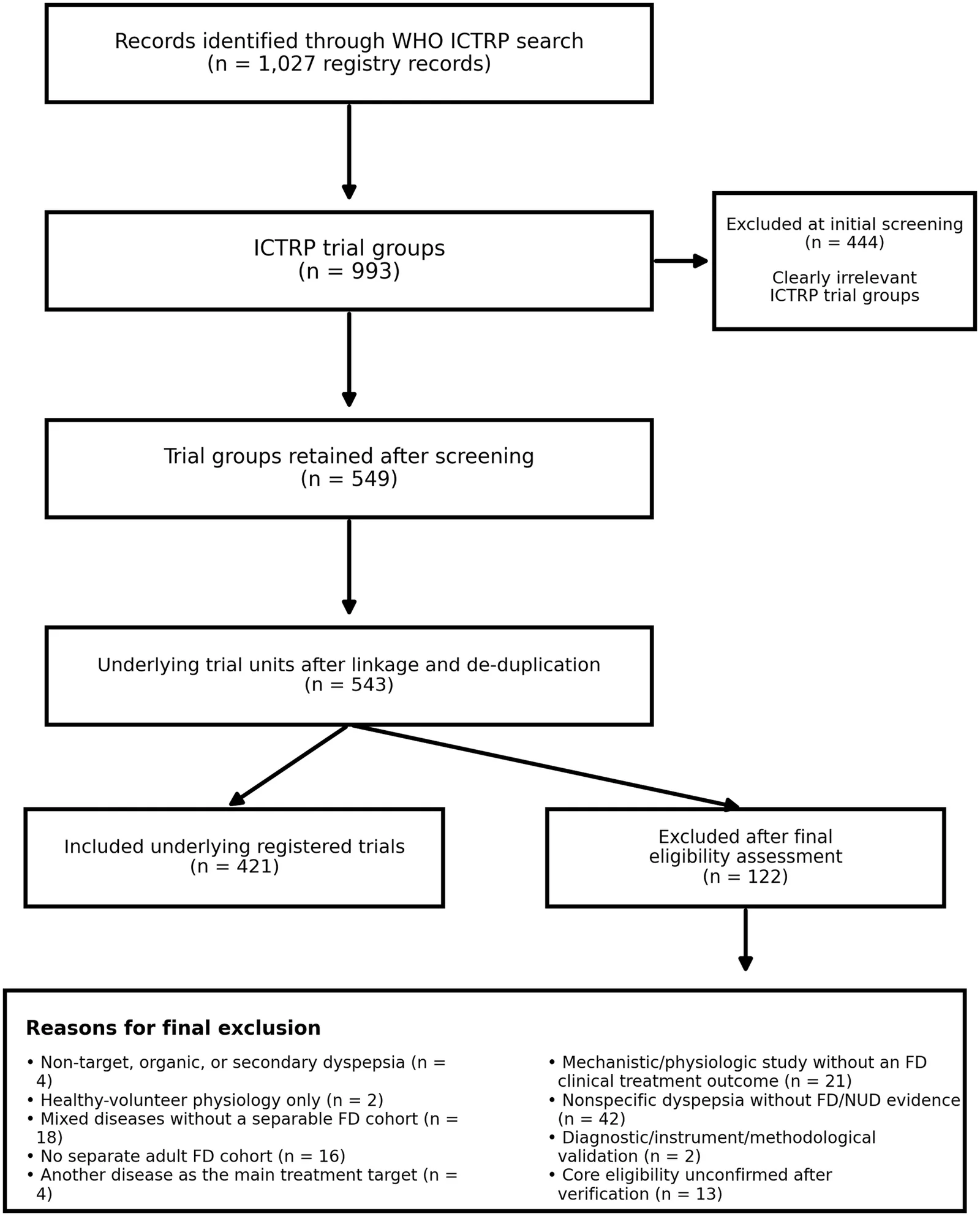
Flow diagram of trial identification, screening, linkage, de-duplication, eligibility assessment, and inclusion. The ICTRP search yielded 1,027 records in 993 trial groups; 421 underlying registered trials were included.

### 3.2 Registration characteristics

The 421 included trials were first registered between 2005 and 2026. The largest share was registered during 2021–2026: 191 trials (45.4%), compared with 59 (14.0%) during 2005–2010, 57 (13.5%) during 2011–2015, and 114 (27.1%) during 2016–2020. The highest annual count was in 2025, with 51 registrations (12.1% of the included set).

The earliest linked registration was most often from ClinicalTrials.gov (157 trials, 37.3%), followed by ChiCTR (60, 14.3%), CTRI (48, 11.4%), and IRCT (42, 10.0%). Using the earliest linked registration date, 252 trials (59.9%) were registered before the recorded study start date and 168 (39.9%) after it. Timing was unclear for one trial (0.2%).

Registered study locations were confined to one country or region in 383 trials (91.0%) and spanned multiple countries or regions in 14 (3.3%); locations were not specified in 24 (5.7%). A country or region was identifiable for 397 trials (94.3%). China was the most frequently listed location (120 trials, 28.5%), followed by India (49, 11.6%), Iran (43, 10.2%), and South Korea (23, 5.5%). Belgium, Japan, Thailand, and the United States were each listed in 22 trials (5.2%), followed by the United Kingdom (11, 2.6%) and Germany (9, 2.1%). Multinational trials contributed to more than one location, so country-specific percentages were not mutually exclusive.

A target sample size could be confirmed for 309 trials (73.4%). In these trials, the median was 100 participants (interquartile range, 60–180; range, 5–1,500). For 10 of these 309 trials, later linked records reported different positive values, and the value from the record with the earliest registration date was retained. Of the remaining 112 trials, 90 had no verifiable positive planned target sample size after checking ICTRP and the corresponding source registry; 21 had only a current enrollment figure whose planned or actual status could not be determined; and one had conflicting values on the earliest registration date. Table 1 summarizes registration characteristics.

**Table 1:** Registration characteristics of included underlying registered trials.

Characteristic	Category	Value
Earliest linked registration period	2005–2010	59 (14.0%)
	2011–2015	57 (13.5%)
	2016–2020	114 (27.1%)
	2021–2026	191 (45.4%)
Source registry of earliest linked registration	ClinicalTrials.gov	157 (37.3%)
	ChiCTR	60 (14.3%)
	CTRI	48 (11.4%)
	IRCT	42 (10.0%)
	ITMCTR	25 (5.9%)
	JPRN	22 (5.2%)
	TCTR	20 (4.8%)
	EU Clinical Trials Register	18 (4.3%)
	ISRCTN	15 (3.6%)
	ANZCTR	8 (1.9%)
	Clinical Trials Information System	3 (0.7%)
	NL-OMON	1 (0.2%)
	REPEC	1 (0.2%)
	German Clinical Trials Register	1 (0.2%)
Registration timing relative to recorded study start date	Before recorded study start date	252 (59.9%)
	After recorded study start date	168 (39.9%)
	Unclear	1 (0.2%)
Registered recruitment status	Recruiting	114 (27.1%)
	Not yet recruiting	2 (0.5%)
	Completed	8 (1.9%)
	Not recruiting	283 (67.2%)
	Unknown	10 (2.4%)
	Not specified	4 (1.0%)
Registered study geography	Single-country or region	383 (91.0%)
	Multi-country or region	14 (3.3%)
	Not specified	24 (5.7%)
Target sample size	Confirmed	309 (73.4%)
	Median (IQR)	100 (60–180)
	Range	5–1,500

Percentages use all 421 included underlying registered trials unless stated otherwise. Sample-size statistics describe the 309 trials with a confirmed target sample size. IQR, interquartile range.

Registry-source summaries were based on the source registry of each trial's earliest linked registration; all linked records remained available for verification. ChiCTR, Chinese Clinical Trial Registry; CTRI, Clinical Trials Registry–India; IRCT, Iranian Registry of Clinical Trials; ITMCTR, International Traditional Medicine Clinical Trial Registry; JPRN, Japan Primary Registries Network; TCTR, Thai Clinical Trials Registry; ANZCTR, Australian New Zealand Clinical Trials Registry.

### 3.3 Trial design and intervention characteristics

Randomized allocation was reported in 315 trials (74.8%). Allocation was non-randomized in 15 (3.6%), not applicable in 16 (3.8%), and insufficiently specified in 75 (17.8%). Parallel-group designs were the most common intervention model (324 trials, 77.0%), followed by single-group (34, 8.1%) and crossover designs (17, 4.0%). The intervention model was not specified in 40 trials (9.5%).

Placebo was the most common control type (179 trials, 42.5%), followed by active control (95, 22.6%). Thirty-two trials (7.6%) had no concurrent control, and control type was not specified in 79 (18.8%). Participant masking was reported in 112 trials (26.6%), investigator masking in 98 (23.3%), outcome-assessor masking in 62 (14.7%), and care-provider or interventionist masking in 57 (13.5%). Masking roles were not specified in 143 trials (34.0%).

The most frequent mutually exclusive primary intervention categories were prokinetic drugs (57 trials, 13.5%), acid-suppression drugs (56, 13.3%), plant extracts (55, 13.1%), traditional Chinese medicine formulas or patent medicines (44, 10.5%), and acupuncture or acupoint interventions (42, 10.0%). Other categories included other drugs, central neuromodulators, supplements, dietary interventions, antibiotics or H. pylori eradication, psychological or behavioral interventions, electrical or device-based interventions, probiotics, combination therapies, and other non-pharmacological interventions. Table 2 and Figure 2 present the design and intervention characteristics.

**Table 2:** Trial design and primary intervention characteristics.

Characteristic	Category	*n*	%
Allocation	Randomized	315	74.8
Allocation	Non-randomized	15	3.6
Allocation	Not applicable	16	3.8
Allocation	Not specified	75	17.8
Intervention model	Parallel	324	77.0
Intervention model	Crossover	17	4.0
Intervention model	Single-group	34	8.1
Intervention model	Factorial	5	1.2
Intervention model	Mixed parallel/crossover	1	0.2
Intervention model	Not specified	40	9.5
Control type	Placebo	179	42.5
Control type	Active	95	22.6
Control type	Sham	15	3.6
Control type	Usual care	3	0.7
Control type	Waiting-list/no-treatment	2	0.5
Control type	Multiple control	16	3.8
Control type	No concurrent control	32	7.6
Control type	Not specified	79	18.8
Masking: Participants	Yes	112	26.6
Masking: Participants	No	78	18.5
Masking: Participants	Unclear	88	20.9
Masking: Participants	Not specified	143	34.0
Masking: Care providers/interventionists	Yes	57	13.5
Masking: Care providers/interventionists	No	133	31.6
Masking: Care providers/interventionists	Unclear	88	20.9
Masking: Care providers/interventionists	Not specified	143	34.0
Masking: Investigators	Yes	98	23.3
Masking: Investigators	No	91	21.6
Masking: Investigators	Unclear	89	21.1
Masking: Investigators	Not specified	143	34.0
Masking: Outcome assessors	Yes	62	14.7
Masking: Outcome assessors	No	127	30.2
Masking: Outcome assessors	Unclear	89	21.1
Masking: Outcome assessors	Not specified	143	34.0
Primary intervention category	Drug—Prokinetic	57	13.5
Primary intervention category	Drug—Acid suppression	56	13.3
Primary intervention category	Drug—Plant extract	55	13.1
Primary intervention category	Drug—TCM formula/patent medicine	44	10.5
Primary intervention category	Acupuncture/acupoint	42	10.0
Primary intervention category	Drug—Other	29	6.9
Primary intervention category	Drug—Central neuromodulator	28	6.7
Primary intervention category	Supplement	27	6.4
Primary intervention category	Diet	18	4.3
Primary intervention category	Drug—Antibiotic or H. pylori eradication	16	3.8
Primary intervention category	Psychological/behavioral	13	3.1
Primary intervention category	Electrical/device	11	2.6
Primary intervention category	Probiotic	10	2.4
Primary intervention category	Other non-drug	9	2.1
Primary intervention category	Combination	6	1.4

Percentages use *N* = 421. Each trial was assigned one primary intervention category. TCM, traditional Chinese medicine; H. pylori, Helicobacter pylori.

Masking was classified separately for each explicitly reported role; a trial could report masking for more than one role. Generic blinding labels were not used to infer unreported roles. “Yes” and “No” indicate that role-specific masking status could be determined; “Unclear” that masking was reported but the role assignment could not be resolved; and “Not specified” that no usable masking information was available.

**Figure 2:**
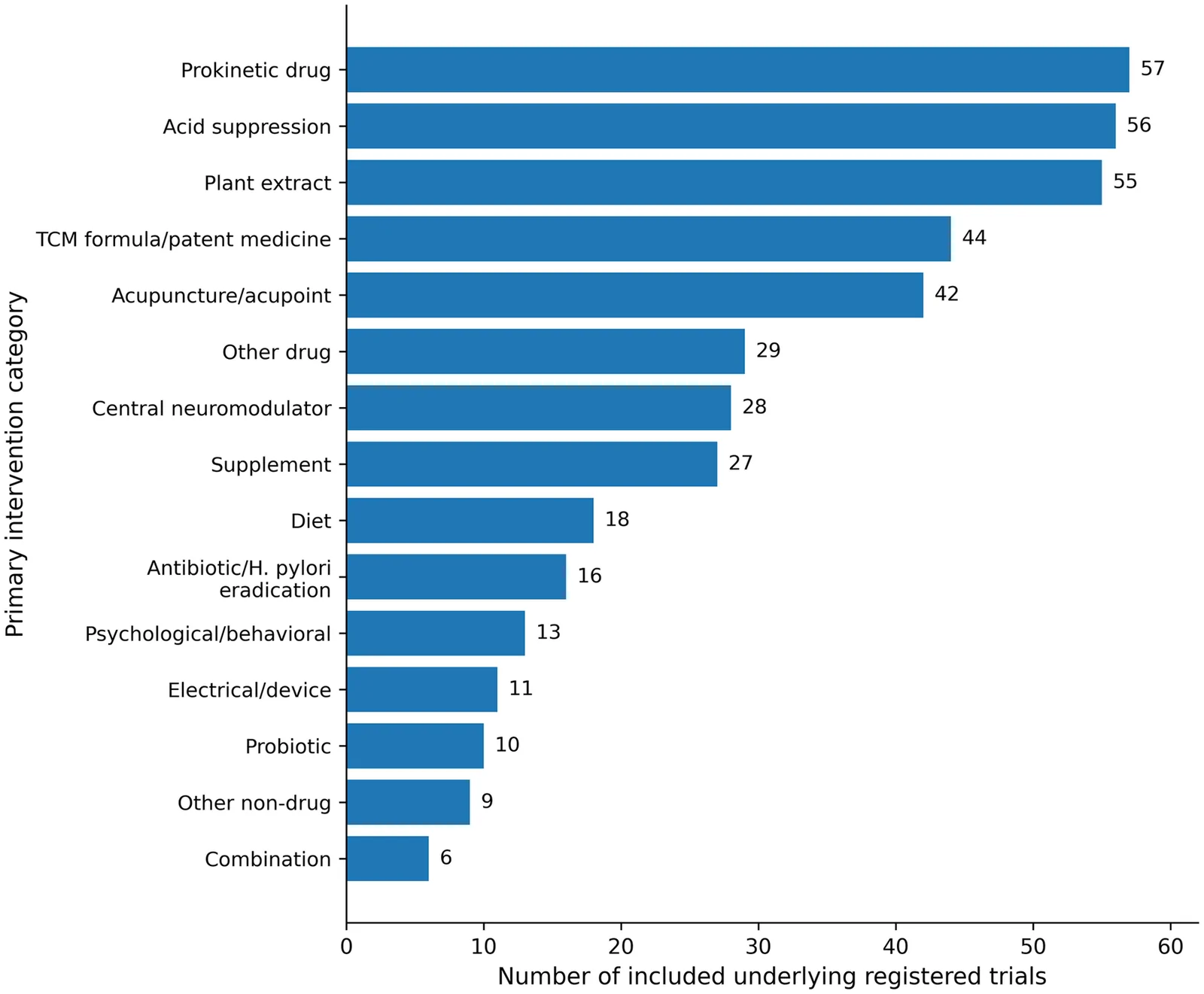
Distribution of mutually exclusive primary intervention categories among included underlying registered trials. Bars show the number of trials in each category. TCM, traditional Chinese medicine.

### 3.4 Diagnostic and eligibility characteristics recorded in registries

An explicit Rome diagnostic version was identified in 278 trials (66.0%). Rome IV was the most frequently specified version (167 trials, 39.7% of all included trials), followed by Rome III (94, 22.3%) and Rome II (17, 4.0%). No explicit Rome version was identified in 143 trials (34.0%).

Registry information was sufficient to classify endoscopy-related reporting in 243 trials (57.7%): endoscopy was required in 161 (38.2%), conditionally required in 6 (1.4%), and no endoscopy requirement was mentioned in 76 (18.1%). The remaining 178 trials (42.3%) had insufficient information for classification. The “not mentioned” category describes the registry record and does not establish that endoscopy was unnecessary or was not performed.

H. pylori eligibility criteria were identifiable in 107 trials (25.4%), including 84 requiring H. pylori negativity and 12 requiring positivity. GERD overlap criteria were identifiable in 89 trials (21.1%), and IBS overlap criteria in 71 (16.9%). Table 3 gives the detailed distributions.

**Table 3:** Diagnostic, eligibility, outcome, and registry-content characteristics.

Characteristic	Category	*n*	%
Rome diagnostic criteria	Rome II	17	4.0
Rome diagnostic criteria	Rome III	94	22.3
Rome diagnostic criteria	Rome IV	167	39.7
Rome diagnostic criteria	No explicit Rome version identified	143	34.0
Upper GI endoscopy	Required	161	38.2
Upper GI endoscopy	Conditionally required	6	1.4
Upper GI endoscopy	Not mentioned	76	18.1
Upper GI endoscopy	Insufficient registry information for classification	178	42.3
H. pylori eligibility handling	Positive required	12	2.9
H. pylori eligibility handling	Negative required	84	20.0
H. pylori eligibility handling	Positive allowed	8	1.9
H. pylori eligibility handling	No restriction	3	0.7
H. pylori eligibility handling	No explicit or resolvable rule identified	314	74.6
GERD overlap handling	Unconditionally excluded	29	6.9
GERD overlap handling	Conditionally excluded	55	13.1
GERD overlap handling	Overlap allowed or required	5	1.2
GERD overlap handling	No explicit or resolvable rule identified	332	78.9
IBS overlap handling	Unconditionally excluded	42	10.0
IBS overlap handling	Conditionally excluded	20	4.8
IBS overlap handling	Overlap allowed or required	9	2.1
IBS overlap handling	No explicit or resolvable rule identified	350	83.1
Explicit registry-content indicators	Intervention duration explicitly identified	149	35.4
Explicit registry-content indicators	Post-intervention follow-up duration explicitly identified	15	3.6
Explicit registry-content indicators	Safety/adverse-event outcome explicitly identified	103	24.5
Explicit registry-content indicators	Primary outcome specified	417	99.0
Primary outcome domain(s)	FD symptoms	374	88.8
Primary outcome domain(s)	Physiological or mechanistic	61	14.5
Primary outcome domain(s)	QoL	37	8.8
Primary outcome domain(s)	Psychological	48	11.4
Primary outcome domain(s)	Safety	15	3.6
Primary outcome domain(s)	Adherence	4	1.0
Primary outcome domain(s)	Feasibility	7	1.7
Primary outcome domain(s)	Other	3	0.7
Primary outcome domain(s)	Not specified	4	1.0
Primary FD symptom instrument	Named instrument	166	39.4
Primary FD symptom instrument	Unnamed/descriptive symptom assessment	208	49.4
Primary FD symptom instrument	No primary FD symptom outcome	47	11.2

Percentages use *N* = 421. Primary-outcome domains are not mutually exclusive and may sum to more than 100%. GI, gastrointestinal; GERD, gastroesophageal reflux disease; IBS, irritable bowel syndrome; QoL, quality of life.

Missing or unresolved registry information does not establish that a procedure or assessment was absent during the trial. For endoscopy, “Not mentioned” indicates that relevant eligibility information was available but no requirement was stated; “Insufficient registry information for classification” indicates that the record could not be classified. For GERD and IBS, “Overlap allowed or required” also includes records that explicitly stated no exclusion for the overlap condition. The 103 trials with a safety or adverse-event outcome include outcomes registered as either primary or secondary; 15 trials classified safety as a primary outcome domain. “No primary FD symptom outcome” denotes that instrument classification was not applicable.

### 3.5 Registered outcomes and availability of key registry content

Primary outcomes were specified in 417 trials (99.0%), with FD symptoms included in 374 (88.8%). Other primary-outcome domains were physiological or mechanistic measures in 61 trials (14.5%), psychological outcomes in 48 (11.4%), quality of life in 37 (8.8%), safety in 15 (3.6%), feasibility in 7 (1.7%), adherence in 4 (1.0%), and other outcomes in 3 (0.7%). These domains were not mutually exclusive.

Of the 374 trials with a primary FD symptom outcome, 166 named a specific symptom instrument and 208 used an unnamed or descriptive symptom assessment. These categories represented 39.4% and 49.4% of all included trials, respectively. The remaining 47 trials (11.2%) had no primary FD symptom outcome to which an instrument could be assigned. A safety or adverse-event outcome was identified in the registry information for 103 trials (24.5%).

An explicit intervention duration was identified in 149 trials (35.4%), and an explicit post-intervention follow-up duration in 15 (3.6%). Table 3 summarizes outcome and registry-content characteristics; Figure 3 shows selected registration characteristics and information availability.

**Figure 3:**
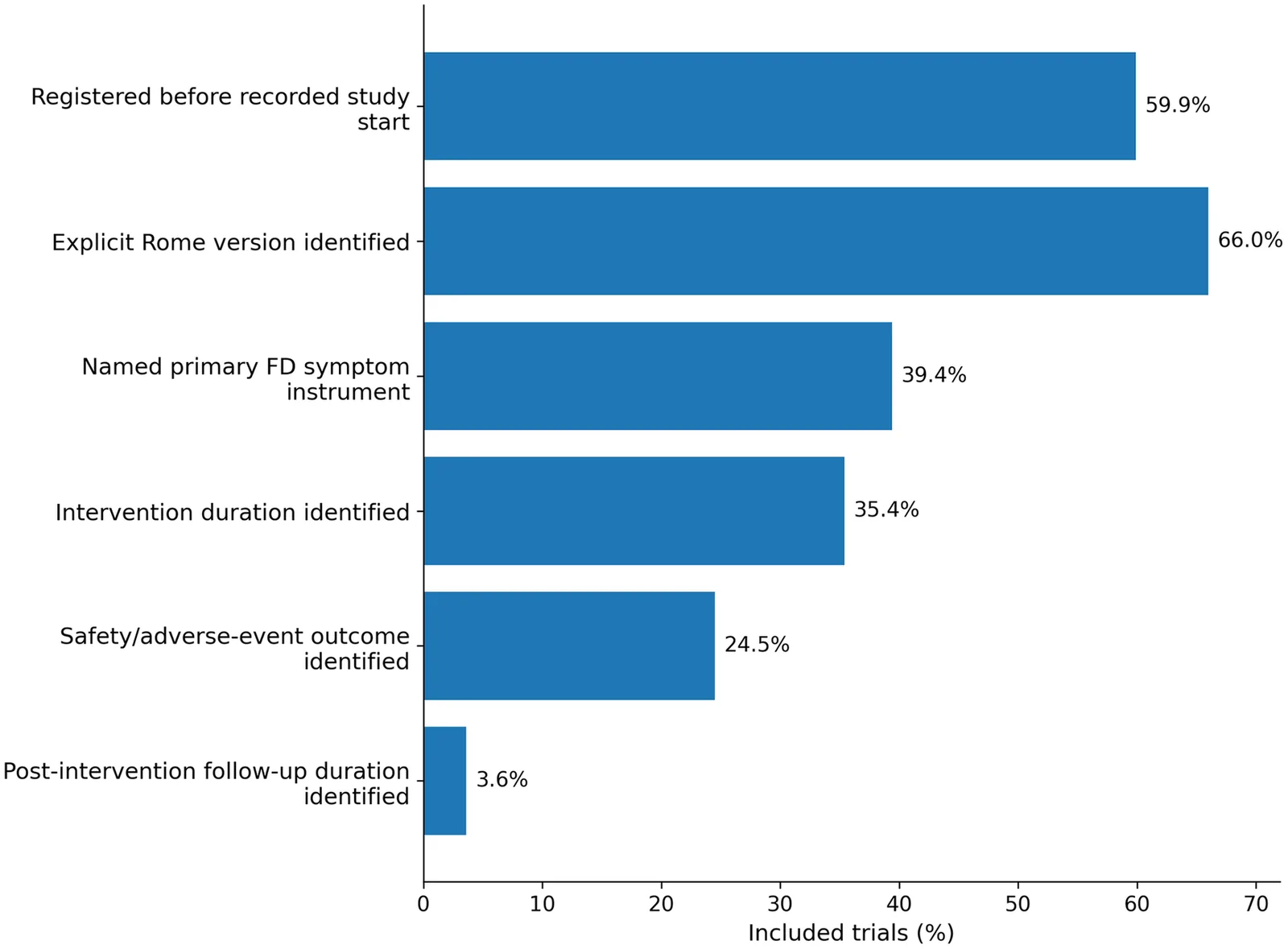
Selected registration characteristics and availability of key registry information among the 421 included underlying registered trials. Bars show the percentage of included trials for each indicator.

## 4 Discussion

### 4.1 Principal findings

This review mapped 421 registered therapeutic trials in adults with FD. The trials covered a range of drug and non-drug interventions, and most described randomized allocation and parallel-group designs. Primary outcomes were usually recorded and commonly included FD symptoms. Other information was less consistently available, particularly the symptom instrument used, safety or adverse-event outcomes, intervention duration, and post-intervention follow-up. The findings show variation in registered designs as well as gaps in the information needed to interpret them.

### 4.2 Registration practice, geographic scope and intervention landscape

Registration activity was greater in recent years, although the latest period ends at the search date in 2026. For 39.9% of trials, the earliest linked registration date followed the recorded study start date. The latter does not necessarily mark first-patient enrollment, so this comparison should be interpreted cautiously. When registration followed the recorded study start date, the registry entry was less useful as a record of methods and outcomes specified in advance.

Most trials listed study locations in a single country or region (91.0%), and only 3.3% listed multiple countries or regions. China was the most frequently represented location (28.5%), followed by India (11.6%) and Iran (10.2%). These records indicate geographic concentration and limited multinational participation within the included set. They do not establish the geographic distribution of all FD research or the locations where recruitment ultimately occurred.

No single intervention category dominated. Prokinetics, acid-suppression drugs, plant extracts, traditional Chinese medicine formulas or patent medicines, and acupuncture or acupoint interventions were common. The range of treatments is consistent with approaches discussed in previous reviews (2, 3). These proportions describe trials identified through the ICTRP search. They may reflect registry coverage and regional registration practices, and should not be read as the distribution of all published or unregistered FD research, or as evidence of comparative efficacy.

### 4.3 Diagnostic and eligibility characteristics

Rome IV was the most frequently named diagnostic framework. The presence of Rome II and Rome III is expected in a set of trials spanning more than two decades; an earlier version may have been appropriate when a trial was designed. More difficult to interpret are the 34.0% of trials for which no explicit Rome version was identified. A named diagnostic framework helps readers assess whether study populations are comparable (4, 5). Its absence from the record does not, by itself, establish that the diagnostic approach was inappropriate.

Endoscopy requirements were variably documented. Some records required endoscopy, others made it conditional, and many either did not mention it or lacked sufficient information for classification. These categories describe what could be established from registry entries, not whether endoscopy was performed. The role of endoscopy must also be considered in relation to patient selection and clinical context (15, 16). Registry entries would be easier to interpret if they explained how organic disease was excluded and when endoscopy was required.

Eligibility rules for H. pylori, GERD overlap, and IBS overlap were often unclear or absent from registry records. These features matter because infection status and overlapping disorders may affect symptom presentation, patient selection, and treatment response (17–19). Explicit criteria would help readers understand the populations studied and judge the applicability of trial findings.

### 4.4 Trial design and masking characteristics

Randomized allocation and parallel-group designs were the predominant approaches. Placebo and active controls were also common. However, allocation was insufficiently specified in 17.8% of trials and control type in 18.8%. Missing basic design information makes it harder to interpret a trial from its registry entry, even when other fields are complete.

Masking requires particular care when comparing registry entries. Generic labels do not consistently identify which roles were masked, which is why we relied on explicit role descriptions. Feasibility also differs among drug trials and trials of acupuncture, dietary modification, psychological therapy, or device-based interventions. A low frequency of recorded masking for a given role cannot, on its own, establish poor trial quality. Stating the roles directly would make the intended design clearer and allow readers to consider masking in the context of the intervention.

### 4.5 Registered outcomes, symptom instruments, safety and follow-up

Although FD symptoms were usually included among primary outcomes, only 39.4% of all trials named a primary FD symptom instrument; 49.4% used an unnamed or descriptive assessment, and 11.2% had no primary FD symptom outcome. Previous reviews have reported substantial variation in FD symptom instruments and patient-reported outcome measures (8, 9). Naming an instrument makes the assessment easier to identify, although this alone does not establish its suitability or measurement properties. Clear descriptions of symptom measures would help readers compare outcomes across trials and plan subsequent evidence synthesis.

Safety or adverse-event outcomes were explicitly identified in 24.5% of trials, intervention duration in 35.4%, and post-intervention follow-up duration in 3.6%. These figures describe registry information and do not indicate that safety monitoring or follow-up was absent in the remaining trials. For a chronic and recurrent condition such as FD (2, 3), clear registration of intervention duration and post-intervention observation helps define the intended scope of therapeutic evaluation.

### 4.6 Implications for future FD trials and registry reporting

Future registrations should state diagnostic and eligibility criteria, masking roles, and outcome instruments clearly. They should also distinguish intervention duration from post-intervention follow-up and state planned safety or adverse-event outcomes clearly. Earlier registration would strengthen the registry's value as a record of intended methods and outcomes. These changes would make trial entries easier to interpret and compare.

Greater consistency in registration does not require identical trial designs. The appropriate population, comparator, masking arrangements, and observation period depend on the intervention and research question. The aim is to make these choices explicit. International collaboration may also broaden the geographic representation of FD trials, given the small proportion with registered locations in multiple countries.

### 4.7 Strengths and limitations

Using WHO ICTRP gave access to records from multiple registries. Linking records at the trial level reduced the risk of counting the same trial more than once, while source-registry checks helped resolve missing or inconsistent information. Screening and trial-level eligibility assessment were performed independently by two reviewers, and trial-level data were independently extracted by two reviewers, with adjudication when needed. Missing or unresolved information was retained as a separate reporting category, and masking was classified according to explicitly reported roles rather than inferred from generic blinding labels.

Using the most recent verifiable source-registry record captures the information available at data verification and may be closer to a trial's later recorded configuration. However, entries can be amended during or after trial conduct, including changes to outcomes; the latest record cannot establish what was prospectively registered before enrollment (20, 21). This review primarily describes the latest verifiable registry information for fields resolved under this rule; it does not provide a systematic reconstruction of the original prerecruitment versions for all 421 trials.

The review is restricted to registered therapeutic trials and does not cover unregistered studies or the complete published evidence base. Registry entries describe registered information and may differ from what was ultimately done. Fields vary across platforms, and records can be incomplete, outdated, or amended after initial registration; registered locations may also differ from actual recruitment sites. Dependence on ICTRP terminology and indexing may have led to missed trials. ICTRP aggregates registry-supplied registration data sets searchable in English and links to the full original source-registry records. Additional local-language information or differently indexed fields in individual source registries may therefore not have been retrieved by the ICTRP-only search (14). Source-registry checks for records already identified through ICTRP were not separate discovery searches. In addition, no review protocol was prospectively registered or published, although an operational protocol was finalized before the WHO ICTRP search, screening, trial linkage, and data extraction. We did not appraise risk of bias or methodological quality and did not evaluate treatment efficacy. The results should therefore be used as a descriptive account of registered characteristics.

## 5 Conclusion

The 421 registered FD trials covered a range of pharmacological and non-pharmacological interventions. Primary outcomes were usually specified and commonly symptom-focused, but records varied in their descriptions of diagnostic and overlap criteria, masking roles, symptom instruments, safety or adverse-event outcomes, intervention duration, and post-intervention follow-up. Earlier registration and clearer reporting of these elements would make trial designs easier to interpret and support comparisons across studies.

## Data Availability

The original contributions presented in the study are included in the article/Supplementary Material, further inquiries can be directed to the corresponding author.

## Supplementary material

Supplementary Protocol S1 operational protocol and decision rules.

Supplementary Appendix S2 complete registry search and record-management documentation.

Supplementary Checklist S3 PRISMA-ScR reporting checklist.

Supplementary Checklist S4 PRISMA-S search-reporting checklist.

Supplementary Dataset S5 complete final trial-level analytic dataset for all 421 included underlying registered trials, with identifiers, source and linkage information, inclusion decisions, analysis classifications, and missing or unresolved codes.
